# Supplementary material for: Insights into the inhibition of type I-F CRISPR-Cas system by a multifunctional anti-CRISPR protein AcrIF24
Source: Nat Commun. 2022 Apr 11;13:1931. doi: 10.1038/s41467-022-29581-1 (PMC9001735; doi:10.1038/s41467-022-29581-1)
Supplement: Supplementary file 1 — Supplementary Information [file 41467_2022_29581_MOESM1_ESM.pdf]

Supplementary Information

Insights into the inhibition of type I-F CRISPR-Cas system by a multifunctional anti-CRISPR protein AcrIF24

Lingguang Yang, Laixing Zhang, Peipei Yin, Hao Ding, Yu Xiao, Jianwei Zeng, Wenhe Wang, Huan Zhou, Qisheng Wang, Yi Zhang, Zeliang Chen, Maojun Yang, Yue Feng

Corresponding Author: Yue Feng
Supplementary Tables 1-3
Supplementary Figures 1-13

Supplementary Table 1 Data collection and refinement statistics

	AcrIF24ΔMD
**Data collection**	
Space group	$P6_122$
Cell dimensions	
a, *b*, *c* (Å)	77.689, 77.689, 145.121
α, β, γ (°)	90.00, 90.00, 120.00
Resolution (Å)	50-2.10 (2.18-2.10)*
R_{sym} or R_{merge} (%)	21.0 (73.2)
$I/\sigma(I)$	16.8 (5.5)
Completeness (%)	99.4 (96.8)
Redundancy	5.0 (3.9)
**Refinement**	
Resolution (Å)	37.52-2.10
No. reflections	15709
R_{work} / $R_{free}^{\#}$	0.2135/0.2293
No. atoms	1421
Protein	1271
Ligand/ion	0
Water	150
B factors	33.34
Protein	33.19
Ligand/ion	
Water	34.54
R.m.s. deviations	
Bond lengths (Å)	0.004
Bond angles (°)	0.55
Ramachandran plot (%)	
Favored	99.37
Allowed	0.63
Outliers	0.00

*For each structure one crystal was used. *Values in parentheses are for highest-resolution shell.

Supplementary Table 2 Statistics of the molecular weight of each peak calculated by SLS

Proteins	Ratio	Peak	molecular weight by SLS (kDa)*	Figs.
AcrIF24	-		AcrIF24 (2): 51.8 ± 1.8	Supplementary Fig. 4b
Csy complex	-		Csy complex (1): 357.4 ± 2.6	Supplementary Fig. 4b
Csy-AcrIF24 complex	-		Csy complex (2):AcrIF24 (2): 769.7 ± 2.8	Supplementary Fig. 4b
Csy:AcrIF24	5:1	peak 1	Csy complex (2):AcrIF24 (2): 776.0 ± 2.4	Fig. 3b
		peak 2	Csy complex (1): 354.6 ± 1.3	
Csy:AcrIF24	1:1	peak 1	Csy complex (2):AcrIF24 (2): 770.6 ± 2.2	Fig. 3b
		peak 2	Csy complex (1):AcrIF24 (2): 427.1 ± 1.2	
Csy:AcrIF24	1:5	peak 1	Csy complex (2):AcrIF24 (2): 770.6 ± 2.2	Fig. 3b
		peak 2	Csy complex (1):AcrIF24 (2): 418.3 ± 1.7	
		peak 3	AcrIF24 (2): 49.8 ± 0.5	

*The theoretical molecular weight of the Csy complex and AcrIF24 is 350.4 and 24.96 kDa, respectively.

Supplementary Table 3 Cryo-EM data collection and model refinement

	Csy-AcrIF24 (EMD-31185, PDB 7ELM)	Csy-AcrIF24-dsDNA$_{SP}$ (EMD-31186, PDB 7ELN)	Csy-AcrIF24-dsDNA$_{NS}$ (EMD-32440, PDB 7WE6)
**Data collection and processing**			
Microscope		Tian Krios TEM (Thermo Fisher)	
Camera		Gatan K3 Summit direct electron detector	
Energy filter		Gatan GIF Quantum, 20eV slit	
Magnification (calibrated)		81,000 ×	
Voltage		300 kV	
Defocus range (μm)		-1.3 - -2.3	
Total electron exposure (e-/Å2)		50	
Exposure rate (e-/pixel/sec)		20	
Pixel size (Å)	1.0742		1.1
**Reconstruction**			
Software		RELION	
Micrographs used	2,374	2,793	4,294
Total extracted particles	1,122,799	933,567	1,079,487
Total refined particles	150,969	188,384	474,421
Symmetry applied		*C1*	
Map resolution (Å, FSC=0.143)	2.88	3.02	3.20
Local resolution range (Å)		2.4-4.0	
**Real space refinement**			
Software		phenix_real_space_refine in the PHENIX	
Model resolution (Å, FSC=0.5)	3.07	3.19	3.50
Model composition			
Non-hydrogen atoms	47399	48906	48325
Protein residues	6201	6209	6209
DNA/RNA	120	172	164
CC (box)	0.85	0.86	0.85
B factor of protein residues (mean)	66.09	78.97	150.12
B factor of nucleotide (mean)	138.93	212.26	216.33
R.M.S deviations			
Bond lengths (Å)	0.005	0.007	0.007
Bond angles (Å)	0.772	0.826	0.908
**Validation**			
Molprobity Score	1.76	1.74	2.39
Clashscore	6.36	6.53	6.53
Rotamer outliers (%)	0.40	0.58	5.94
C-beta deviations	0	0	0
CaBLAM outliers (%)	3.76	3.39	3.88
Ramachandran plot statics			
Favored (%)	93.91	94.39	93.23
Allowed (%)	6.05	5.54	6.76
Outliers (%)	0.03	0.06	0.02

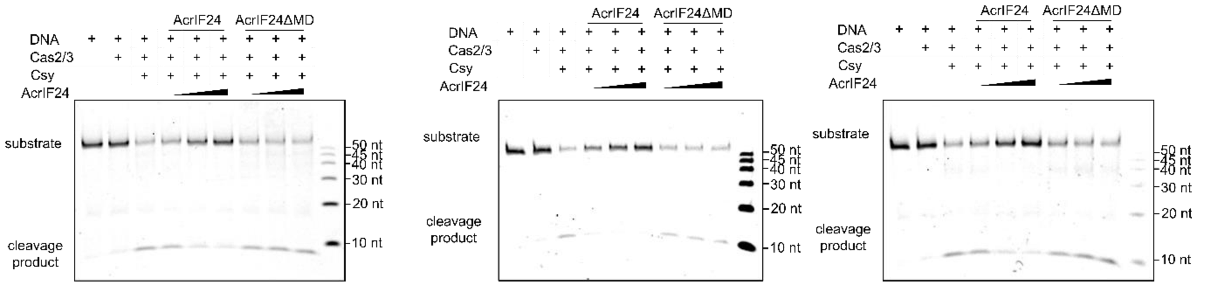

Supplementary Fig. 1 Inhibition of AcrIF24 and AcrIF24ΔMD on the *in vitro* cleavage activity of type I-F CRISPR-Cas system.

Raw data for the calculation of the inhibition abilities of AcrIF24 and AcrIF24ΔMD towards the type I-F CRISPR-Cas system *in vitro* in Fig. 1a.

a

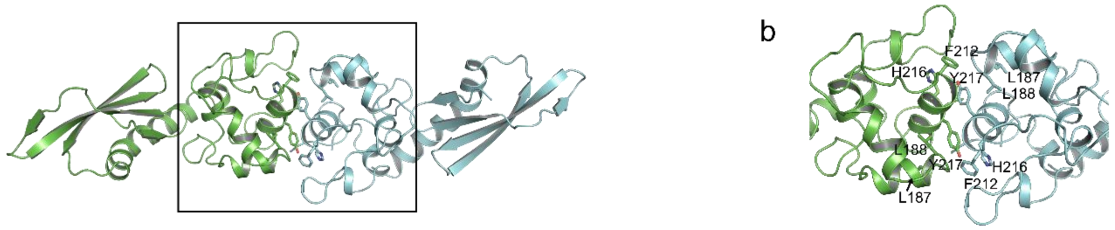

c

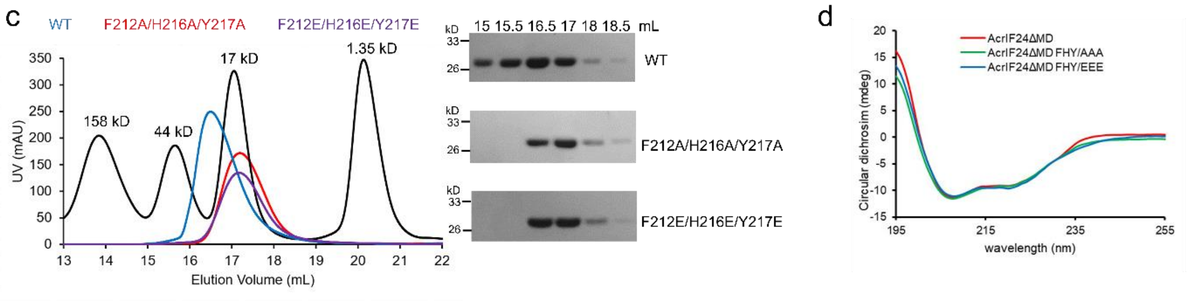

e

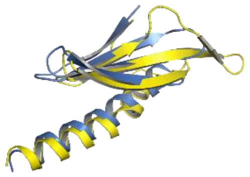

f

No.	PDB Chain	Z score	RMSD (Å)	Aligned residues	Molecule name
1	7c0a-B	6.5	2.6	65	Anti-crispr-associated protein aca1
2	7cq9-A	6.1	2.4	59	Uncharacterized protein aca2
3	4yba-B	5.9	2.1	60	Regulatory protein c
4	5jaa-A	5.8	2.5	61	Antitoxin iga-2
5	3f51-A	5.7	1.7	55	CLP gene regulator (clgr)
6	3op9-A	5.6	2.8	59	Pli0006 protein
7	2ox6-C	5.4	1.7	57	Hypothetical protein so3848
8	3omt-A	5.3	1.9	54	Uncharacterized protein
9	6jq1-B	5.2	2.4	56	Transcriptional regulator, xre family
10	4o8b-A	5.2	1.6	55	Uncharacterized protein

g

No.	PDB Chain	Z score	RMSD (Å)	Aligned residues	Molecule name
1	6d7k-H	6.1	2.3	60	Methane monooxygenase hydroxylase, mmox1
2	5z38-C	5.2	2.8	64	Cest protein
3	5n9j-A	5.2	2.4	59	Mediator of rna polymerase ii transcription subunit
4	5fl0-A	5.1	4.3	66	GP37
5	2na4-A	4.6	3	63	Curli production assembly/Transport component csg
6	6f1t-K	4.5	2.6	64	ARP1 actin related protein 1 homolog a
7	6ozj-B	4.4	2.7	52	Endonuclease V
8	2ygk-A	4.3	6	68	Nura
9	2eab-A	4.3	4.2	62	Alpha-fucosidase
10	2o2a-B	4.2	2.8	61	Hypothetical protein gbs1413

h

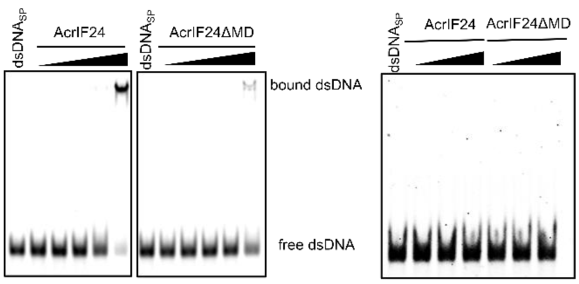

Supplementary Fig. 2 AcrIF24 is a dimer.

(a) The AcrIF24 dimer is shown in cartoon. The interface of two AcrIF24 protomers is marked in a box.

(b) Close-up view of the interface of the two AcrIF24 protomers. Residues involved in the interactions are shown in sticks.

(c) Gel filtration chromatography of AcrIF24ΔMD and its F212A/H216A/Y217A and F212E/H216E/Y217E mutants, along with the gel filtration standard. Fractions were analyzed by SDS-polyacrylamide gel electrophoresis (SDS-PAGE) and are numbered according to their elution positions in the right panel.

(d) Circular dichroism (CD) spectra of AcrIF24ΔMD and its F212A/H216A/Y217A and F212E/H216E/Y217E mutants used in Supplementary Fig. 2c.

(e) Structural superimposition between AcrIF24^{NTD} and the auxiliary protein of soluble methane monooxygenase hydroxylase, MMOD (PDB: 6D7K). AcrIF24^{NTD} is colored in yellow as in Fig. 1b. MMOD is colored in marine.

(f-g) DALI search results of AcrIF24-NTD (f) and AcrIF24-CTD (g) with PDB chain, Z score,

RMSD, number of aligned residues, and molecule name.

(h) EMSA used to test the binding affinities of AcrIF24 or AcrIF24ΔMD for $dsDNA_{SP}$. Reactions were performed as in Fig. 2c.

(i) EMSA used to test the binding affinities of AcrIF24 or AcrIF24ΔMD for $dsDNA_{SP}$. Reactions were performed with 0.04 μM 54-bp $dsDNA_{SP}$. AcrIF24 or AcrIF24ΔMD was added with concentrations of 0.16, 0.32, and 0.64 μM following the order indicated by the black triangles.

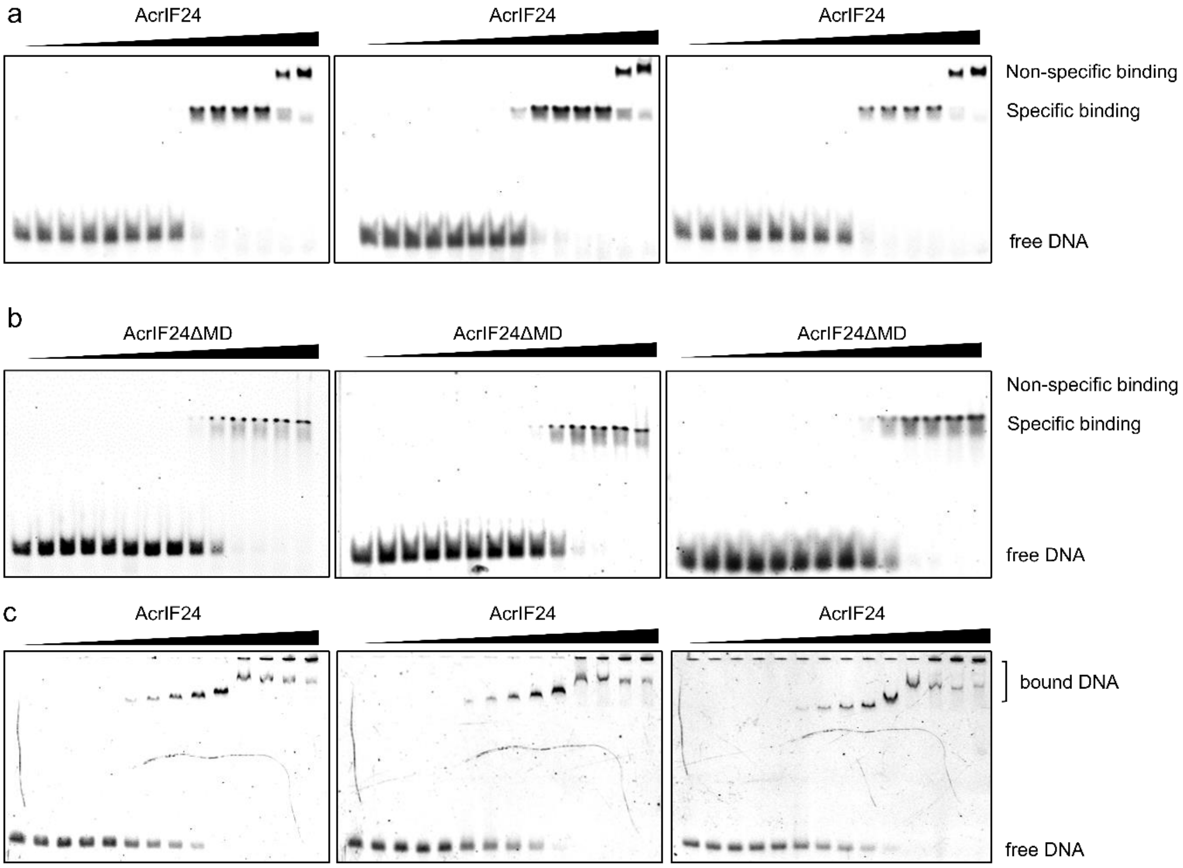

Supplementary Fig. 3 Binding of AcrIF24 or AcrIF24ΔMD to IR23-24.

(a-b) Raw data for the calculation of the binding affinity to the IR23-24 sequence of AcrIF24 (a) or AcrIF24ΔMD (b) in Fig. 2b. Specifically, AcrIF24 or AcrIF24ΔMD was incubated in a concentration gradient (0, 0.01, 0.05, 0.1, 0.5, 1, 5, 10, 50, 100, 500, 1000, 5000, 10000 nM) with 20 nM of 23-bp IR23-24.

(c) Raw data for the calculation of the binding affinity to $dsDNA_{NS}$ of AcrIF24. Specifically, AcrIF24 was incubated in a concentration gradient (0, 0.01, 0.1, 0.5, 1, 2, 3, 4, 6.275, 12.5, 50, 100, 200, 400 μM) with 20 nM of 54-bp $dsDNA_{NS}$.

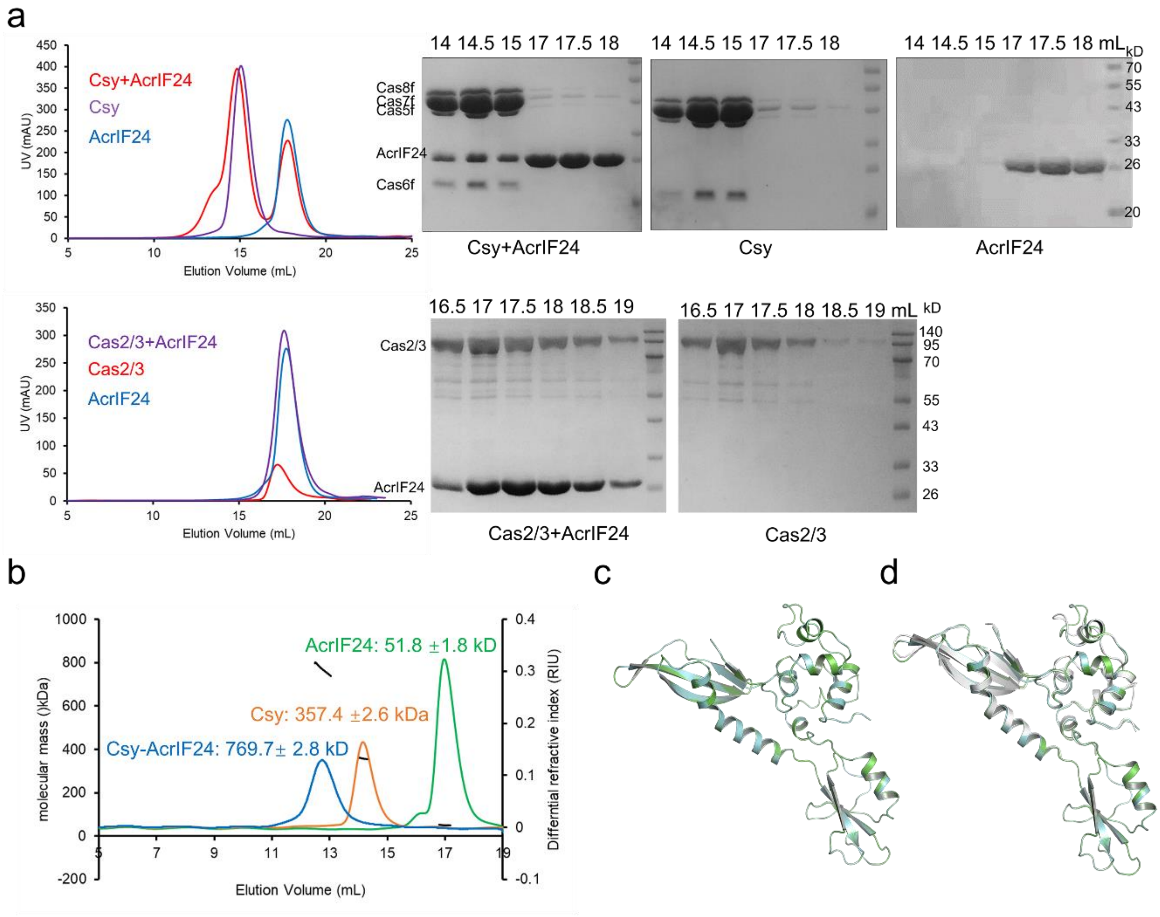

Supplementary Fig. 4 AcrIF24 interacts with the Csy complex to form a heterotetrameric complex.

(a) Gel filtration chromatography of Csy complex, Cas2/3, AcrIF24, and the mix of Csy complex or Cas2/3 and AcrIF24. Fractions were analyzed by SDS-PAGE and are numbered according to their elution positions in the right panel.

(b) Static light scattering (SLS) studies of Csy complex, AcrIF24 and co-expressed Csy-AcrIF24 complex. The calculated molecular weights of the main peaks of the three profiles are shown above the peaks.

(c) The alignment of two AcrIF24 protomers in cryo-EM structure shown in cartoon.

(d) The alignment of AcrIF24 protomers in cryo-EM structure (cyan and green) and that in crystal (gray).

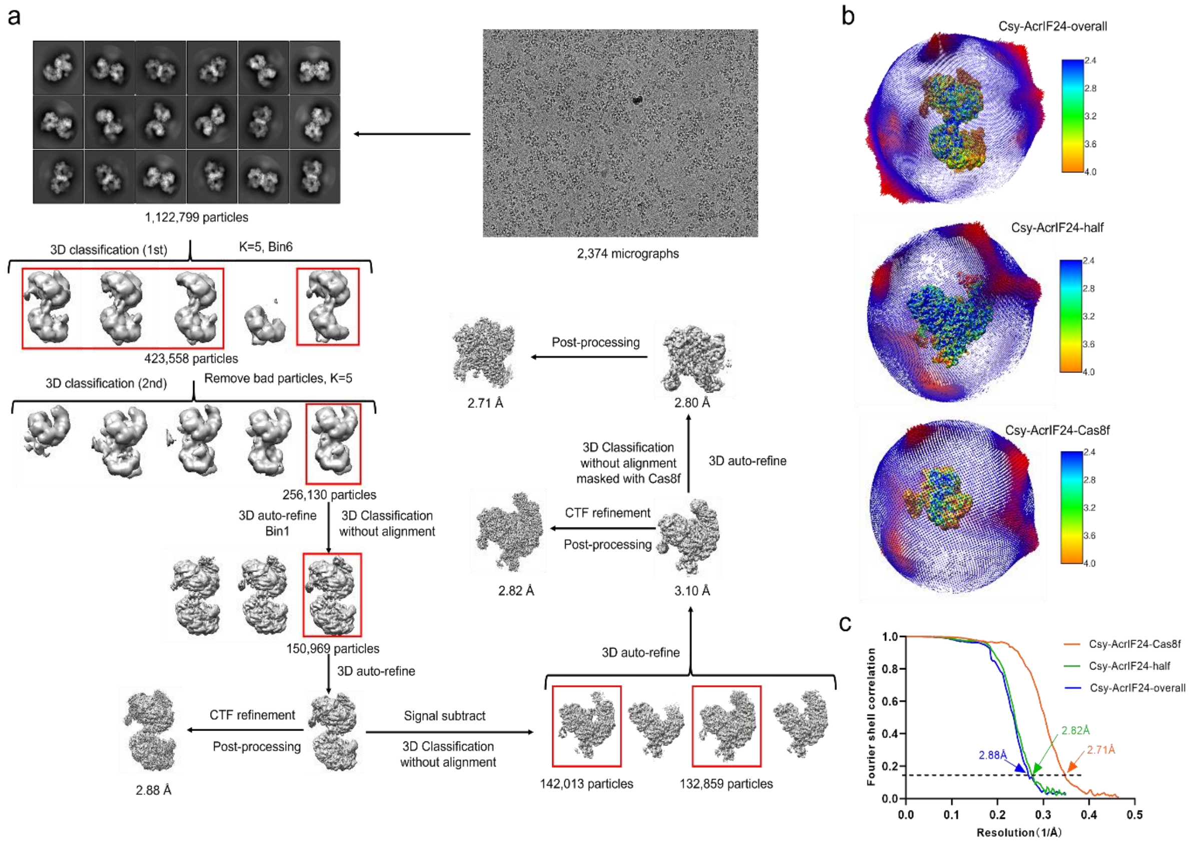

Supplementary Fig. 5 Cryo-EM image processing for the Csy-AcrIF24 complex.

(a) Representative processing pipeline includes a representative raw cryo-EM micrograph, 2D class averages, 3D classification and refinement of cryo-EM particles.

(b) Local resolution estimation and Euler angle distribution of the map of the overall Csy-AcrIF24 (Csy-AcrIF24-overall), Csy-AcrIF24 protomer (Csy-AcrIF24-half) and the Cas8f part of Csy-AcrIF24 protomer (Csy-AcrIF24-Cas8f), respectively.

(c) Plot of the global FSC indicates an average resolution of 2.88 Å for the overall Csy-AcrIF24 structure, 2.82 Å for the Csy-AcrIF24 protomer and 2.71 Å for the Cas8f part of Csy-AcrIF24 protomer.

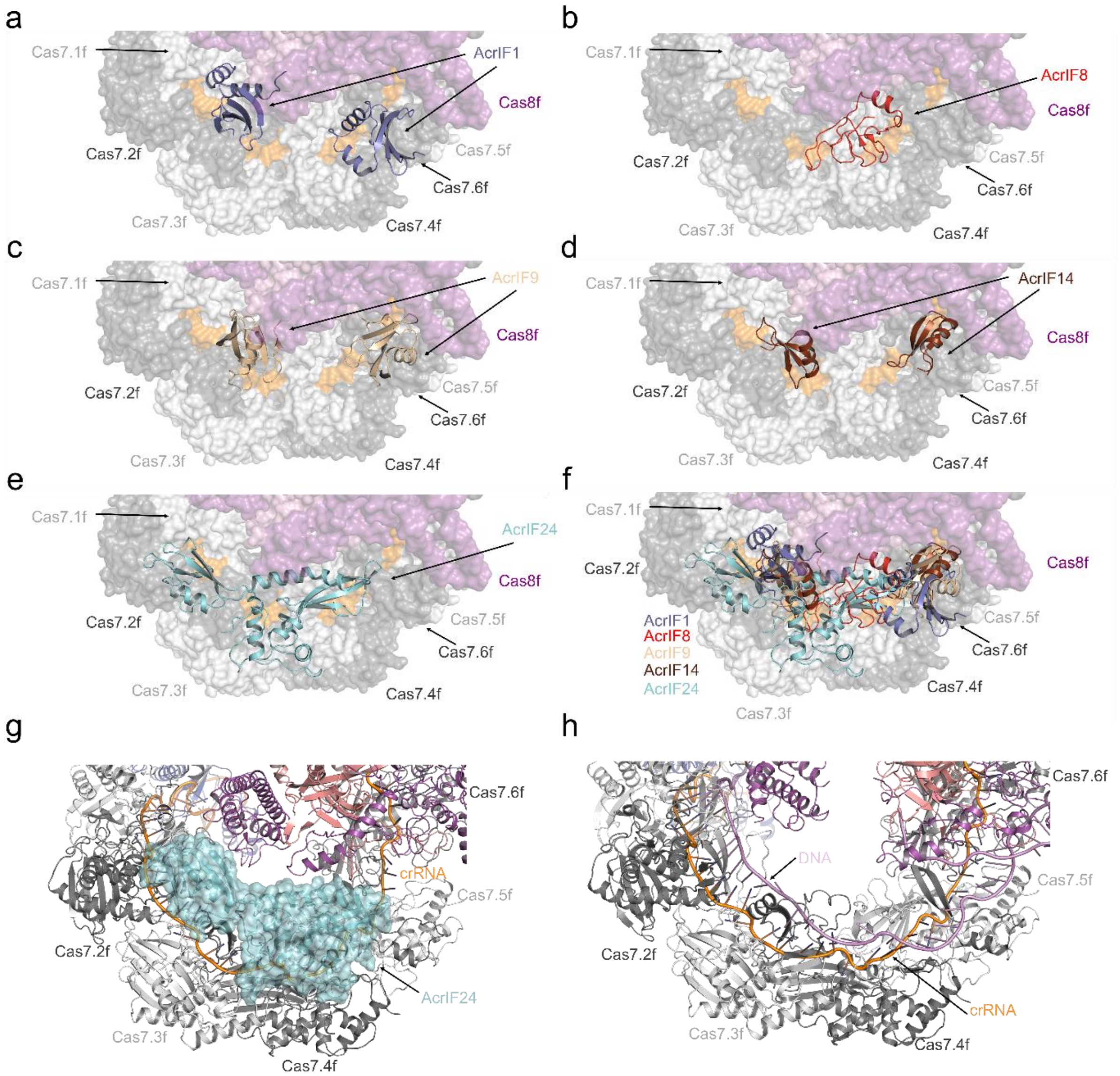

Supplementary Fig. 6 AcrIF24 interacts with almost the whole Cas7f backbone to compete with target dsDNA.

(a-e) Atomic structure of Csy-AcrIF1 (a; PDB code: 6B46), Csy-AcrIF8 (b; PDB code: 6VQW), Csy-AcrIF9 (c; PDB code: 6VQV), Csy-AcrIF14 (d; PDB code: 7JZZ), and Csy-AcrIF24 (e) in cartoon representation with each subunit of Csy complex colored as in Fig. 3d. AcrIF1, AcrIF8, AcrIF9, AcrIF14 and AcrIF24 are colored in slate, red, wheat, chocolate and cyan, respectively.

(f) Structural superimposition of the structures shown in a-e.

(g) Atomic structure of Csy-AcrIF24 in cartoon representation with each subunit colored as in Fig. 3d. The AcrIF24 is shown in cartoon and surface.

(h) Atomic structure of Csy-dsDNA$_{SP}$ (PDB code: 6NE0) in cartoon representation with each subunit colored as in Fig. 3d. The dsDNA$_{SP}$ is colored in violet.

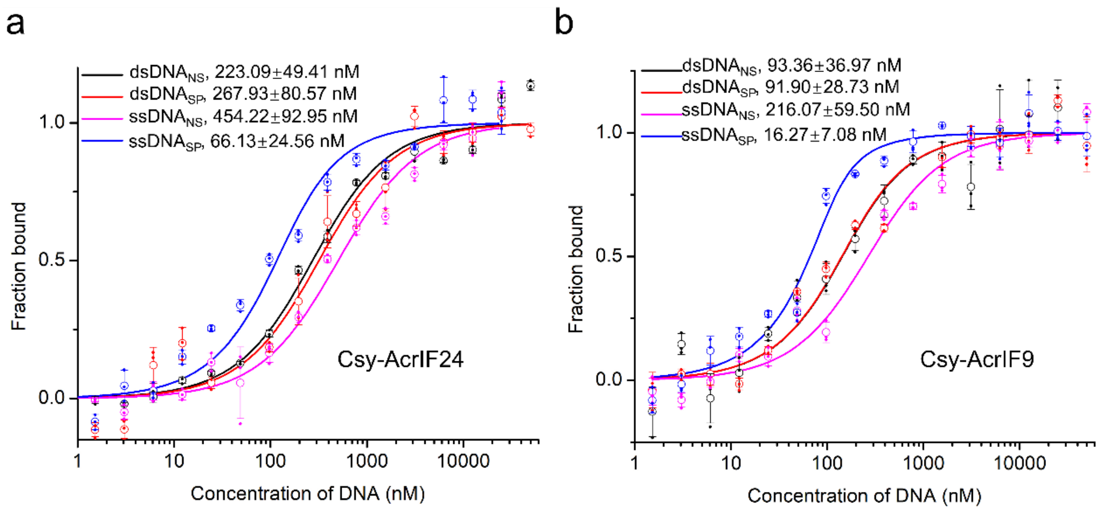

Supplementary Fig. 7 MST assays of the binding of four types of DNA molecules to Csy-AcrIF24 (a) and Csy-AcrIF9 (b)

Means are indicated by circles and individual values from three independent experiments are shown with their markers. Error bars represent SD. Binding curves and K_D values are also shown.

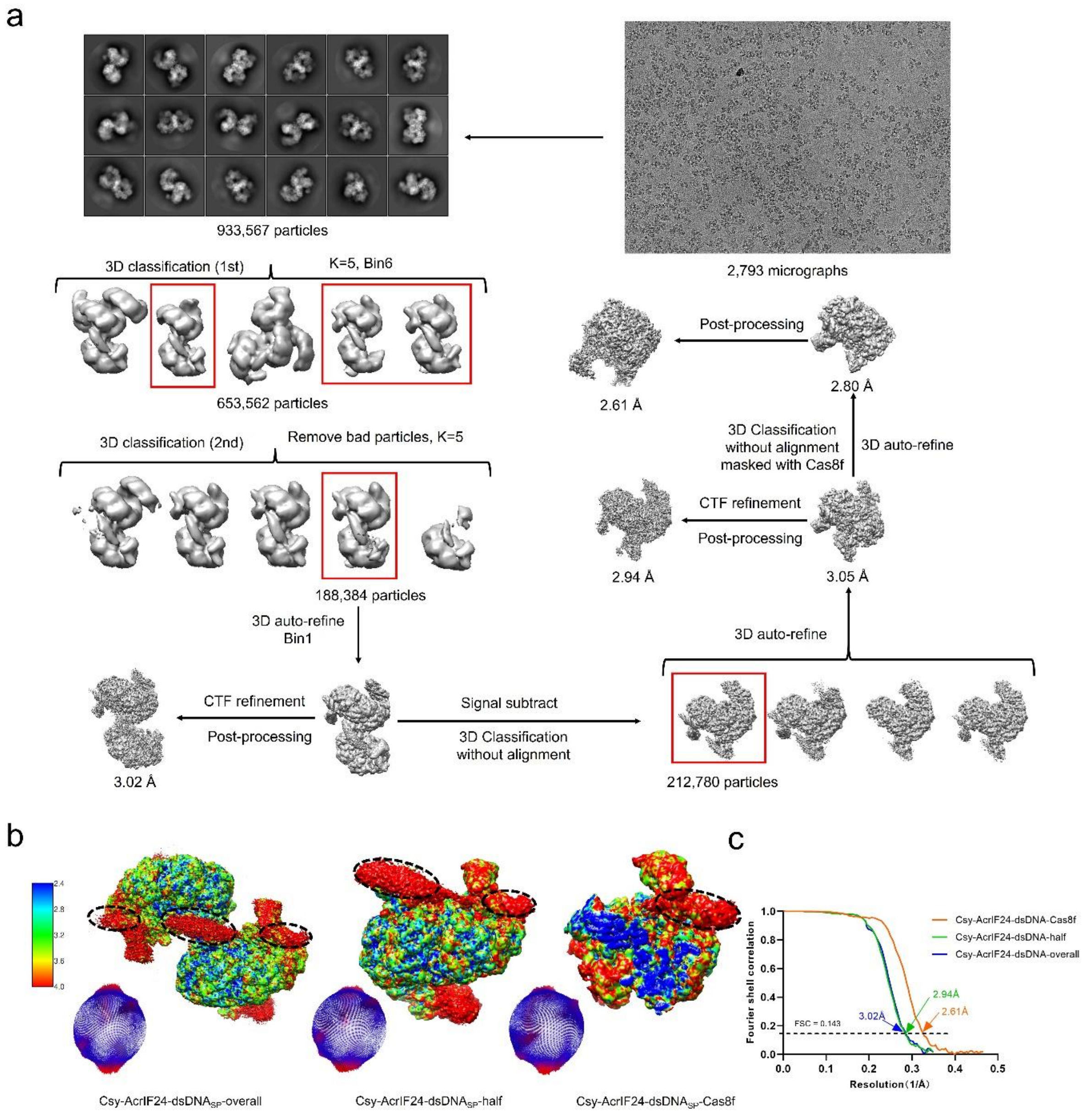

Supplementary Fig. 8 Cryo-EM image processing for the Csy-AcrIF24-dsDNASP complex.
(a) Representative processing pipeline includes a representative raw cryo-EM micrograph, 2D class averages, 3D classification and refinement of cryo-EM particles.
(b) Local resolution map and Euler angle distribution of the map of the overall Csy-AcrIF24-dsDNASP (Csy-AcrIF24-dsDNASP-overall), Csy-AcrIF24-dsDNASP protomer (Csy-AcrIF24-dsDNASP-half) and the Cas8f part of Csy-AcrIF24-dsDNASP protomer (Csy-AcrIF24-dsDNASP-Cas8f), respectively. Maps corresponding to bound DNA molecules are marked in circles.
(c) Plot of the global FSC indicates an average resolution of 3.02 Å for the overall Csy-AcrIF24-dsDNASP structure, 2.94 Å for the Csy-AcrIF24-dsDNASP protomer and 2.61 Å for the Cas8f part of Csy-AcrIF24-dsDNASP protomer.

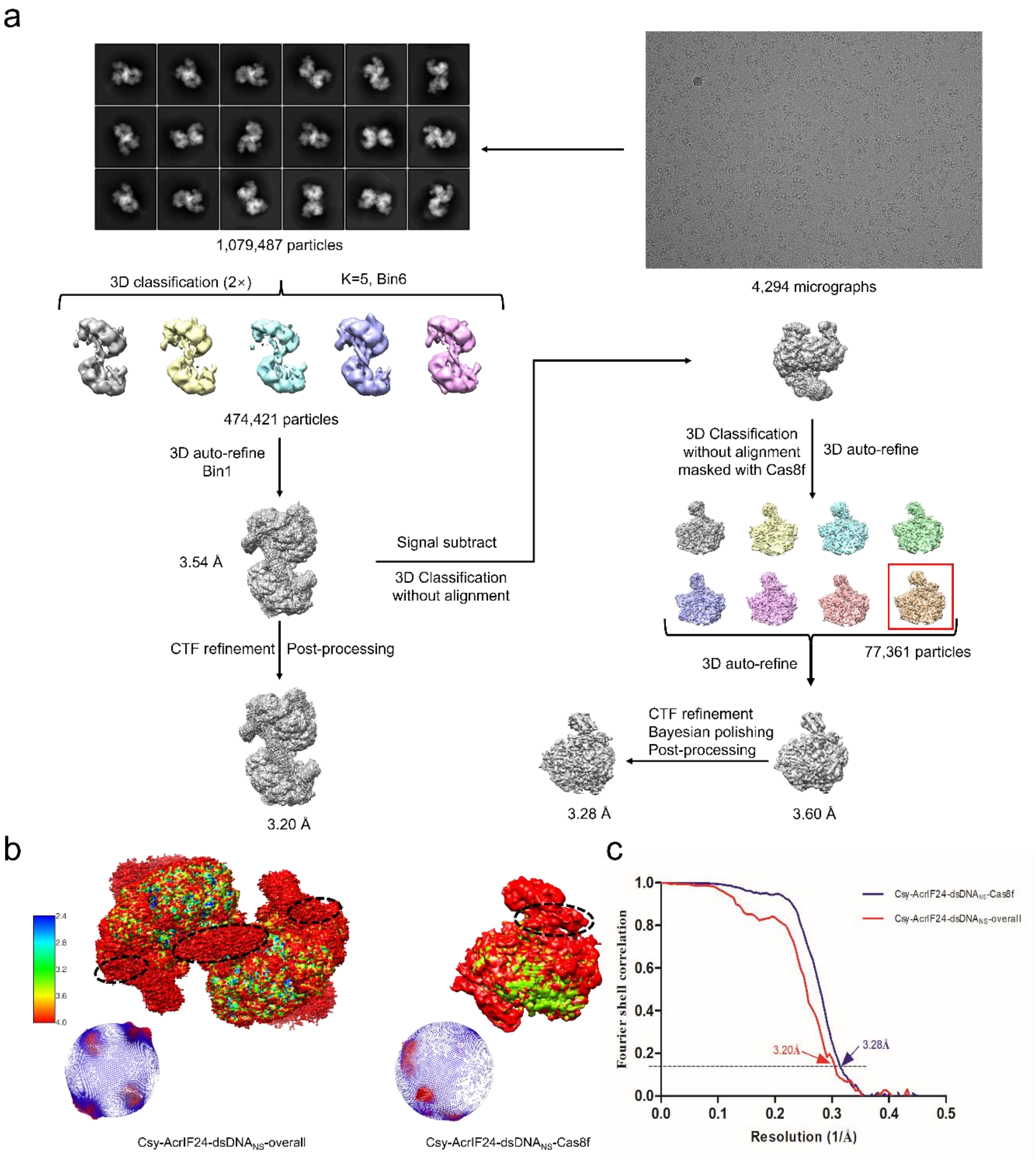

Supplementary Fig. 9 Cryo-EM image processing for the Csy-AcrIF24-dsDNA$_{NS}$ complex. (a) Representative processing pipeline includes a representative raw cryo-EM micrograph, 2D class averages, 3D classification and refinement of cryo-EM particles.
(b) Local resolution map and Euler angle distribution of the map of the overall Csy-AcrIF24-dsDNA$_{NS}$ (Csy-AcrIF24-dsDNA$_{NS}$-overall) and the Cas8f part of Csy-AcrIF24-dsDNA$_{NS}$ protomer (Csy-AcrIF24-dsDNA$_{NS}$-Cas8f), respectively. Maps corresponding to bound DNA molecules are marked in circles.
(c) Plot of the global FSC indicates an average resolution of 3.02 Å for the overall Csy-AcrIF24-dsDNA$_{SP}$ structure, 2.94 Å for the Csy-AcrIF24-dsDNA$_{SP}$ protomer and 2.61 Å for the Cas8f part of Csy-AcrIF24-dsDNA$_{SP}$ protomer.

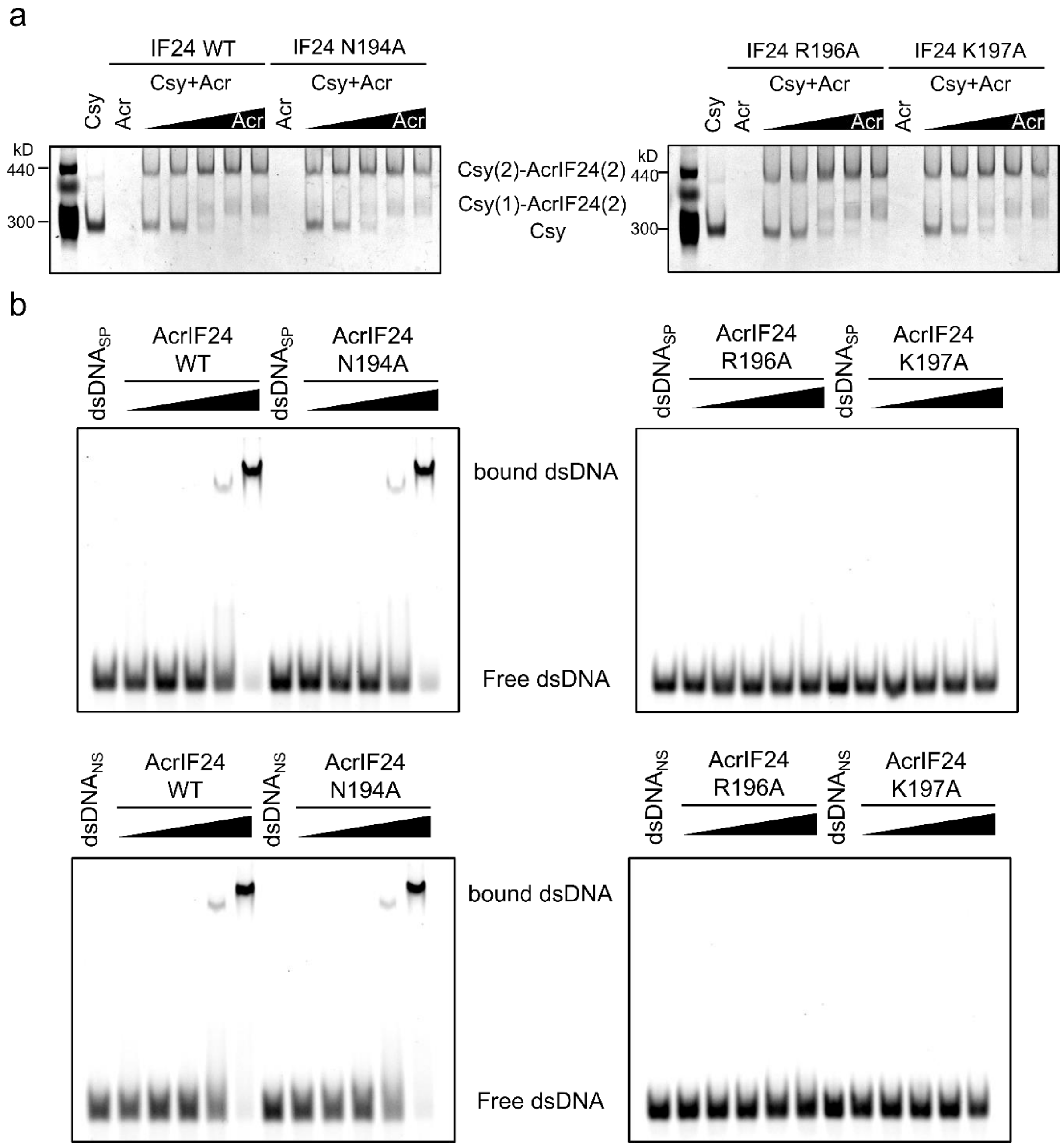

Supplementary Fig. 10 AcrIF24 mutants lacking induction ability of non-specific DNA binding fail to interact with dsDNA, but still interact with the Csy complex.

(a) Native gel used to test the binding between the Csy complex and AcrIF24 or its mutants. Reactions were performed with 0.32 μM Csy complex and AcrIF24 concentrations of 0.16, 0.20, 0.24, 0.28, and 0.32 μM following the order indicated by the black triangle. The gel was stained with Coomassie blue staining.

(b) EMSA used to test the binding affinities of AcrIF24 or its mutants for $dsDNA_{SP}$ and $dsDNA_{NS}$. Reactions were performed as in Fig. 2c.

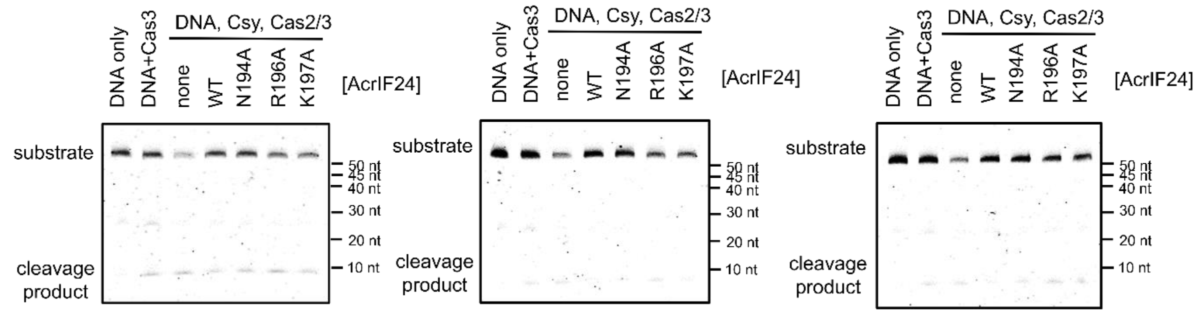

Supplementary Fig. 11 Induction of non-specific DNA binding by AcrIF24 contributes to its inhibitory capacity.

Raw data for the calculation of the inhibition abilities of AcrIF24 or its mutants towards the type I-F CRISPR-Cas system *in vitro* in Fig. 7d. Reactions were performed as in Fig. 1a, except that AcrIF24 or its mutants were added at concentration of 0.16 μM.

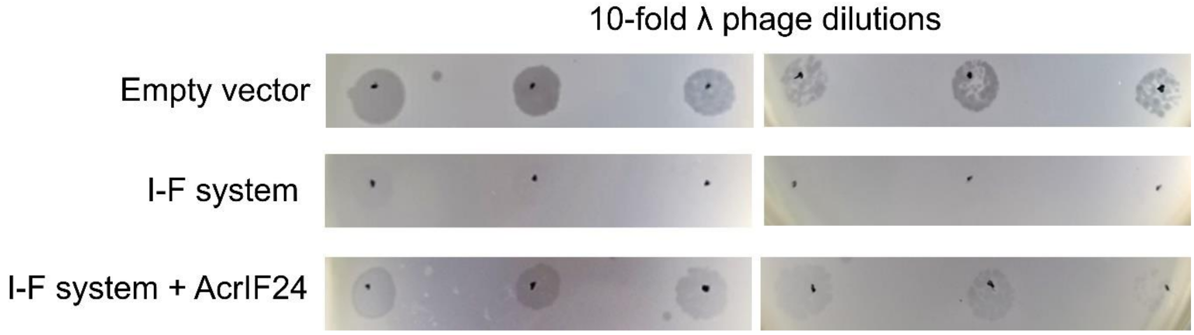

Supplementary Fig. 12 Reconstitution of type I-F CRISPR system in *E. coli* NovaBlue (DE3) cells

400 µL of *E. coli* NovaBlue (DE3) cells grown to $OD_{600\ nm}$ of 0.9 was added into 5 ml of soft LB/agar with 0.25% arabinose and 0.25 mM IPTG and poured onto LB agar plates. 3 µL of 10-fold dilution of λ phage lysate was titrated on the plate surface, and the plates were incubated at 37°C overnight.

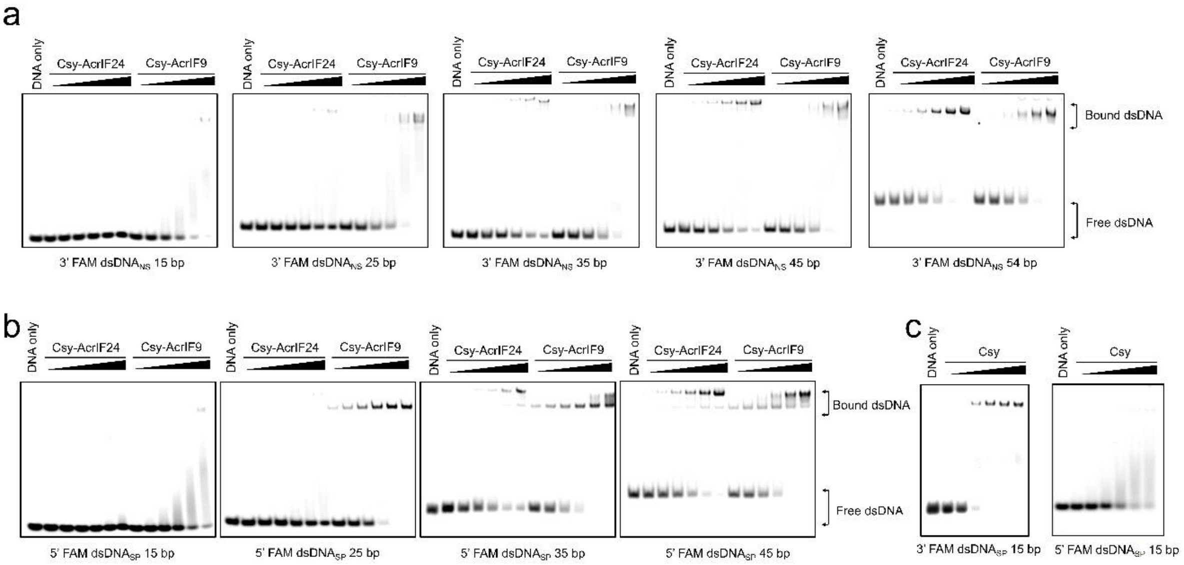

Supplementary Fig. 13 Length of dsDNA matters to the non-specific DNA binding of Csy-AcrIF24, but not Csy-AcrIF9.

(a-b) EMSA used to test the non-specific DNA binding of Csy-AcrIF24 or Csy-AcrIF9 using $dsDNA_{NS}$ with 3' FAM labels (a) and $dsDNA_{SP}$ with 5' FAM labels (b) of different lengths. Reactions were performed as in Fig. 5c.

(c) EMSA used to test the DNA binding ability of Csy complex for 15 bp $dsDNA_{SP}$ with 5' or 3' FAM labels. Reactions were performed as in Fig. 5c.
